# The Interplay between Myocardial Fibrosis, Strain Imaging and Collagen Biomarkers in Adults with Repaired Tetralogy of Fallot

**DOI:** 10.3390/diagnostics11112101

**Published:** 2021-11-13

**Authors:** Konstantina Karali, Kali Makedou, Alexandros Kallifatidis, Matthaios Didagelos, George Giannakoulas, Constantinos H. Davos, Theodoros D. Karamitsos, Antonios Ziakas, Haralambos Karvounis, Stavros Hadjimiltiades

**Affiliations:** 1 First Department of Cardiology, AHEPA Hospital, Faculty of Health Sciences, School of Medicine, Aristotle University of Thessaloniki, St. Kyriakidi 1, 54636 Thessaloniki, Greece; manthosdid@yahoo.gr (M.D.); g.giannakoulas@gmail.com (G.G.); karamits@gmail.com (T.D.K.); tonyziakas@hotmail.com (A.Z.); hkarvounis@gmail.com (H.K.); stavros@otenet.gr (S.H.); 2 Laboratory of Biochemistry, AHEPA General Hospital, Faculty of Health Sciences, School of Medicine, Aristotle University of Thessaloniki, St. Kyriakidi 1, 54636 Thessaloniki, Greece; kalimakedou@gmail.com; 3 Department of Radiology, Cardiovascular Imaging Unit, St. Luke’s Hospital, 55236 Thessaloniki, Greece; alexandros.kallifatidis@yahoo.gr; 4 Cardiovascular Research Laboratory, Biomedical Research Foundation, Academy of Athens, 11527 Athens, Greece; cdavos@bioacademy.gr

**Keywords:** myocardial fibrosis, cardiac magnetic resonance feature tracking (CMR-FT), Galectin-3, Procolagen III, NTproBNP, adults with repaired tetralogy fallot, congenital heart disease

## Abstract

Background: We sought to assess the interplay between right ventricle (RV) fibrosis, biventricular dysfunction based on global longitudinal strain (GLS) analysis, and biomarkers such as Galectin-3 (Gal-3), procollagen type III (PCIII), and NTproBNP. Methods: We studied 35 adult patients with rToF. All patients underwent a cardiac magnetic resonance (CMR) scan including feature tracking for deformation imaging. Blood biomarkers were measured. Results: LGE RV was detected in all patients, mainly at surgical sites. Patients with the highest RV LGE scoring had greater RV dilatation and dysfunction whereas left ventricular (LV) function was preserved. LV GLS correlated with RV total fibrosis score (*p* = 0.007). A LV GLS value of −15.9% predicted LGE RV score > 8 (AUC 0.754 (*p* = 0.02)). Neither RV GLS nor biomarker levels were correlated with the extent of RV fibrosis. A cut-off value for NTproBNP of 145.25 pg/mL predicted LGE RV score > 8 points (AUC 0.729, (*p* = 0.03)). A cut-off value for Gal-3 of 7.42 ng/mL predicted PR Fraction > 20% [AUC 0.704, (*p* = 0.05)]. Conclusions: A significant extent of RV fibrosis was mainly detected at surgical sites of RV, affecting RV performance. CMR-FT reveals subtle LV dysfunction in rToF patients, due to decreased performance of the fibrotic RV. Impaired LV function and elevated NTproBNP in rToF reflect a dysfunctional fibrotic RV.

## 1. Introduction

The improvement in the management of Tetralogy of Fallot (ToF) has led to the extended survival of adult patients with repaired ToF (rToF) [1,2,3,4,5,6], but also to an increased number of patients with heart failure (HF) who may deteriorate and require hospitalization [7,8]. The early diagnosis of HF is a challenge, as the majority of these patients underestimate their symptoms. Myocardial damage resulting from the corrective surgery, the postoperative onset of volume overload or pressure conditions, postoperative conduction disturbances, and ventricular interaction are factors that lead to the development of HF in adult patients with rToF. The neurohormonal profile of patients with congenital heart disease (CHD) is similar to those of HF. Fibrosis biomarkers that have been found to play an important role in heart failure are also increased in adults with rToF [4,5,6].

Furthermore, imaging modalities may add valuable data to risk stratification. Cardiovascular magnetic resonance (CMR) is the gold standard technique for accurate and reproducible noninvasive measurements of biventricular size and function, quantification of valvular regurgitation, and the detection of myocardial fibrosis [9,10,11]. Analysis of myocardial strain with feature tracking (FT), which is a CMR-based method, is a sensitive measure of regional and global ventricular contractile function, and may contribute to risk stratification of the growing rToF population [12,13,14,15].

Therefore, we aimed to assess the following: 1. Detection and distribution of right ventricle (RV) fibrosis and correlations with clinical data; 2. The prognostic role of biomarkers levels in the study population; 3. Analysis of global CMR-FT strain parameters of RV and left ventricle (LV) and their correlation with extent of RV fibrosis and biomarker levels.

## 2. Materials and Methods

The study was conducted at the Adult Congenital Heart Disease Clinic, First Cardiology Department, AHEPA University Hospital, Thessaloniki, Greece. The study protocol was approved by the Institutional Review Board (1/8 January 2012) and all participants provided written informed consent.

### 2.1. Patient Population

We studied 35 consecutive adult patients (21 women, mean age 31 ± 10.8 years) with repaired ToF. Patients with a permanent pacemaker/implantable cardioverter defibrillator (ICD) were excluded, due to contraindication for CMR. All patients had a complete cardiac examination, including past medical history, physical examination, and standard 12-lead ECG, and underwent a CMR study (within 3 months from the clinical examination). Standard 12-lead ECGs were acquired of all patients and the QRS duration was measured manually [15,16,17]. The New York Heart Association (NYHA) class was recorded for all patients [18].

### 2.2. Blood Samples-Biomarkers

Serum was isolated after centrifugation at 4000 rpm for 10 min at 4 °C. Galectin-3 (Gal-3) serum levels were determined using an enzyme-linked immunoabsorbent assay (ELISA) kit (BOSTER Biological Technology, Pleasanton, CA, USA). The inter-assay and intra-assay variation are 8.1% and 6.63%, respectively. Galectin values are expressed in ng/mL. Procollagen type III (PCIII) serum levels were determined using a sandwich ELISA kit (Abbexa Ltd., Cambridge, UK). The inter-assay and intra assay variation are both <10%. PCIII values are expressed in ng/mL. NT-proBNP serum levels were determined on a COBAS 8000 immunoassay analyzer (ROCHE Diagnostics, Mannheim, Germany) with electrochemiluminescence immunoassay (ECLIA), using monoclonal antibodies which recognize epitopes located in the N-terminal part (1–76) of proBNP (1–108). The inter-assay and intra-assay variation are 1.5% and 2.5%, respectively.

### 2.3. CMR Imaging Protocol

CMR imaging was performed with a Siemens Avanto 1.5T MRI scanner, using a body surface coil and a standard imaging protocol, which included ECG-gated steady-state, free-precession cine CMR acquisitions in long-axis and contiguous short-axis cine imaging. CMR variables included RV and LV end-diastolic volume index (EDVi); RV and LV end-systolic volume index (ESVi); RV and LV ejection fraction (EF); RV and LV mass index. The RV free wall below the pulmonary valve was included in the RV mass calculation while trabecular bands on the RV side of the septum were included in blood pool measurements. Pulmonary regurgitation (PR) was estimated with CMR pulmonary artery regurgitant fraction (RF). PR was graded as mild if the RF on CMR was less than 20%, moderate if it was between 20% and 40%, and severe if it was greater than 40%. All measurements were made by an experienced observer, blinded to patient clinical status. Late gadolinium imaging was performed by the same observer with the use of a two-dimensional-segmented phase-sensitive inversion recovery sequence (PSIR), and acquisition optimization for imaging nonischemic myocardial fibrosis, 10 min after intravenous administration of 0.1 mmol/kg of gadobutrol (Gadovist^®^, Bayer Inc. 2920 Matheson Boulevard East Mississauga, Bayer Healthcare, L4W 5R6 Ontario Canada).

**Figure 1:**
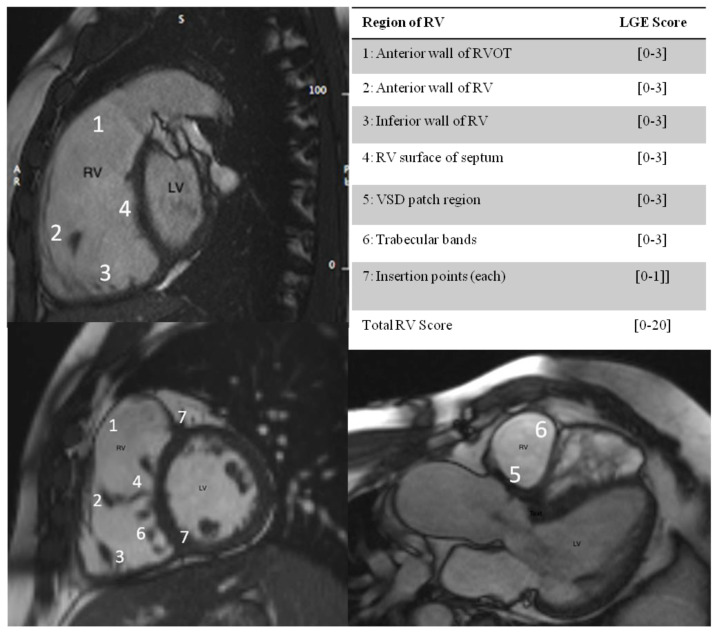
Segmentation system for LGE RV analysis. 1–7: RV segments The division of the RV into 7 segments is shown in slices aligned with the RVOT, the LV outflow tract, and the LV short axis, with maximum LGE score per segment in brackets. [0,1,2,3]: LGE RV SCORE, 0: no linear extent of enhanced myocardium; 1: enhancement of 1 trabeculation; 2: enhancement of 2 to 4 trabeculations; 3: enhancement of more than 4 trabeculations. The insertion points of RV and LV (marked 7) were each scored 0 for absence and 1 for presence of LGE. The maximum score was 20.

For the LGE RV analysis, a segmentation system of RV was utilized as previously described [14]. The RV was divided into 7 segments in slices aligned with the RV outflow tract, the LV outflow tract, and the LV-RV short axis. Segments of RV wall with LGE were scored according to the extent of enhanced myocardium, and expressed as a score out of 20 (Figure 1). Strain analysis: Using specialized software (Circle Cardiovascular Imaging Inc.) from the cine sequences in the longitudinal axis, the global longitudinal strain values for the left and right ventricles were calculated.

### 2.4. Statistical Analysis

All continuous variables are expressed as mean (SD) or median (quartile 1–quartile 3). Continuous variables were analyzed by either 2-sample independent t test or Mann–Whitney test as appropriate. Correlations were assessed by Spearman rank correlation coefficient. Categorical data were analyzed by χ2 test. A nonparametric Kruskal–Wallis test was used to compare different variables in the lower, middle, and upper quartiles of the RV LGE score. Interclass correlation coefficient was used to assess the reproducibility of the RV LGE score. A probability value less than 0.05 was considered statistically significant. The prognostic potential of LV GLS by FT-CMR and biochemical markers Gal-3, PCIII and NTproBNP were tested by ROC curve analysis. Patients with missing data were excluded from the analysis. All data were analyzed with IBM SPSS version 25.0 (Armonk, NY, USA: IBM Corp.).

## 3. Results

### 3.1. Demographic and Clinical Characteristics

The baseline characteristics of the studied cohort are summarized in Table 1.

### 3.2. RV Fibrosis Scoring and Clinical Correlates

RV LGE was detected in all patients at surgical sites, more frequently located in the right ventricular outflow tract (RVOT) scar area (82.8%) and in the site of ventricular septal defect (VSD) patching (51.4%), but also in the anterior wall (65.7%), inferior wall (20%) and RV side of septum (54.2%) and in the RV-LV insertion points (88.5%). There was no LGE in the LV (Supplementary Table S1, Supplementary Figures S1 and S2).

The median RV fibrosis score was 8 points (mean 7.4 ± 2.4, median 8, IQR (6, 10)). We studied the relations of RV fibrosis in two groups of patients: group of patients with low RV score (LGE RV < 8 points) and group of patients with high RV score (LGE RV ≥ 8 points).

The association of LGE RV fibrosis score with clinical markers, CMR indices, and biomarkers is summarized in Table 2.

Patients with above-median RV LGE scores were older and in worst clinical condition based on NYHA Class and rest SatO_2_ compared to patients with low RV fibrosis score (96.5 ± 1.8 versus 97.6 ± 1.1, *p* = 0.05). RV myocardial fibrosis score was associated with RV dilatation and RV dysfunction based on RVEDVi, RVESVi, and RV ejection fraction measurements (Table 2). LV systolic function was worse in the group of patients with high RV fibrosis score compared to low RV fibrosis group. Pulmonary regurgitation severity had a moderate positive correlation with total LGE RV scoring (Table 2). Lowest values of RV EF were observed in patients with high RV score and moderate/severe PR, (Supplementary Figure S3).

### 3.3. RV Fibrosis Scoring and Biomarker Levels in Adults with rToF

Median Gal-3 was 6.1 (5.2, 7.6) ng/mL, median PCIII was 41.8 (38.2, 45.9) ng/mL and median NTproBNP was 143.4 (74.6, 226.5) pg/mL. There was no correlation between collagen biomarkers Gal-3 and PCIII, with NTproBNP.

**Figure 2:**
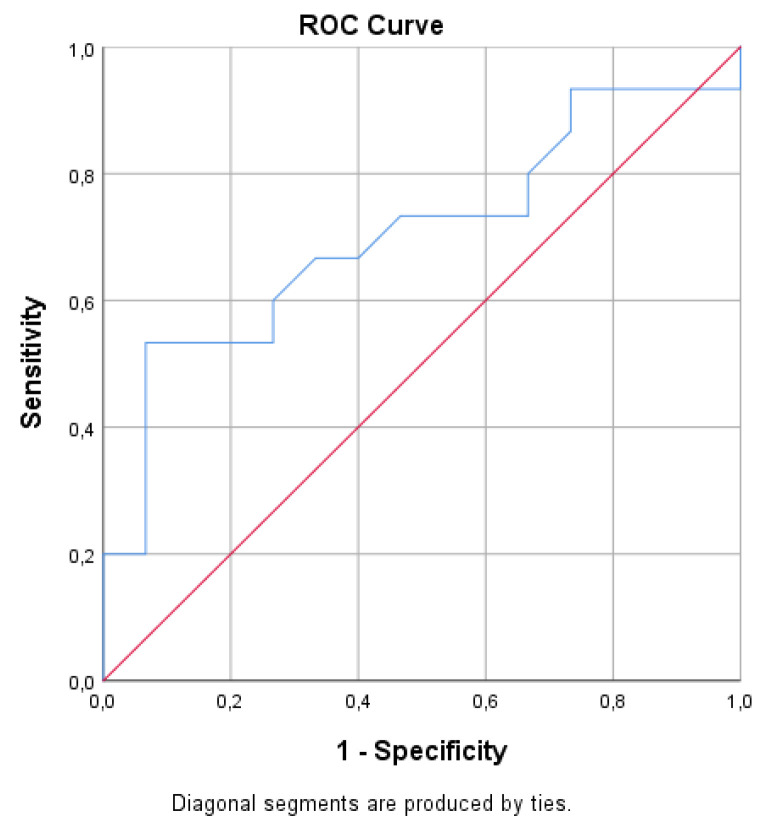
In ROC curve analysis, a cut-off value for Gal-3 of 7.42 ng/mL predicted PR Fraction > 20% with specificity 93% and sensitivity 53% [AUC 0.704, (*p* = 0.05)].

Gal-3 levels were higher in patients with supramedian LGE RV score but did not correlate with total LGE scoring of RV, *p* = 0.21, (Table 2). Gal-3 levels had a significant correlation with moderate/severe PR as estimated by PR Fraction > 20%, *p* < 0.044), (Supplementary Table S2). In ROC curve analysis, a cut-off value for Gal-3 of 7.42 ng/mL predicted PR Fraction >20% with specificity 93% and sensitivity 53% (AUC 0.704, (*p* = 0.05)) (Figure 2).

PCIII levels did not correlate with LGE scoring of RV, (Table 2).

NTproBNP levels were elevated in patients with high RV score, (Table 2).

### 3.4. RV Fibrosis and Cardiac Magnetic Resonance Data with Feature Tracking (CMR-FT) Analysis

The mean value of RV GLS was −20.8 ± 2.47%, with no correlation with high RV scoring, or total RV score (Table 2). The mean value of LV GLS was −17.04 ± 2.61%. LV performance showed a statistically significant difference between fibrosis score groups. LV GLS had a significant correlation with high RV score, *p* = 0.032, and total RV scoring (Table 2).

Biomarker levels did not show any correlation with RV GLS or LV GLS values.

## 4. Discussion

ToF represents the most common form of cyanotic heart disease at birth. Late complications include pulmonary regurgitation, heart failure, and malignant arrhythmias associated with sudden cardiac death (SCD). Areas of fibrosis are detected at the sites of surgery, mainly in the RV outflow tract and the interventricular septum around the ventricular septal defect patch region. Focal fibrosis can be identified using CMR and correlations with both systolic dysfunction and reduced exercise capacity and arrhythmias have been reported [15,16]. Myocardial fibrosis (as estimated by extracellular matrix expansion) has been associated with worse outcomes in heart failure and may even predict mortality in patients with acquired heart disease [19,20].

There is no proven treatment to improve RV function by targeting RV fibrosis, although preclinical studies have shown that Sodium-glucose transport protein 2 inhibitors have also demonstrated a positive effect in cardiac fibrosis [21,22,23].

Our data specifically support an association between RV myocardial fibrosis and impairment of RV function, independently of the first palliation or the highest number of surgeries (Supplementary Table S3). Extended RV fibrosis was observed in patients who were older with residual moderate/severe PR, which suggests that volume overload may have predisposed to myocardial damage and fibrosis in addition to the myocardial surgical scars. Lowest values of RV EF were observed in patients with significant fibrosis of RV and moderate/severe PR (Supplementary Figure S3). Although no LV fibrosis was detected, LV function was affected by high RV fibrosis scoring and RV deterioration. Furthermore, LV long axis displacement by CMR -FT had impaired values in patients with the highest RV fibrosis scoring. Previous studies have shown that patients who underwent corrective surgery at an older age have a higher risk of developing LV dysfunction [24,25,26]. However, our results did not confirm this association.

### 4.1. RV Fibrosis, Biomarker Levels and Prognostic Associations

Gal-3 value of >7.42 pg/mL predicted moderate/severe PR in the studied cohort. In previous studies, Gal-3 has been studied as a biomarker of fibrosis with a prognostic role in HF mortality and rehospitalization and in congenital heart disease patients as a prognostic indicator in risk stratification [21,27,28,29,30,31,32].

Previous studies in patients with rToF, reported that BNP levels are increased and associated with overload conditions and severity of PR [33,34]. NTproBNP levels in our study did not correlate with the severity of PR. Instead, NTproBNP levels correlated with high LGE RV scoring. NTproBNP levels of 145.25 pg/mL predicted an LGE RV score > 8 points. The association between NTproBNP and fibrosis could be mediated via focal dilatation of RV outflow tract. Therefore, NTproBNP could be a sensitive predictive indicator of the functionality of RV, independently of the loading conditions [35,36,37,38].

Levels of Procollagen III and Galectin 3 have been evaluated in HF studies and have been well associated with the prognosis of HF patients with normal cardiac anatomy, with Procollagen III being appreciated as a remodeling index [39]. This fibrosis index in children with congenital shunt lesions was increased due to various hemodynamic disorders, while in children with rToF PCIII levels were related to the degree of cyanosis. Lai et al. reported a good correlation between the mechanical asynchronization of LV and the expression of PCIII in a study of children and young adults with rToF [40]. In our study, which included adults with rToF, PCIII levels had a significant correlation only with LV EF and not with LV GLS or the shunt to repair time.

Biomarker levels did not have a significant correlation with the extent of RV fibrosis in our study. An explanation for the discordance between the extent of RV fibrosis and biomarkers levels may be that in rToF the profibrotic damage-injury occurred at one single time point (at the time of palliation) whereas biomarker levels reflect the neurohormonal activation at the time of assessment. In contrast, in hypertensive patients there is continuous profibrotic damage-injury that constantly induces collagen deposition, and thus biomarker elevation.

### 4.2. Role of Feature Tracking CMR (CMR-FT) in Adults with rToF

Cardiac magnetic resonance feature tracking analysis is progressively establishing its role as an accurate tool for quantitative evaluation of cardiovascular function by directly evaluating myocardial fiber deformation. Feature-tracking derived strain parameters are able to identify subtle myocardial abnormalities before overt clinical manifestation, thus allowing the early diagnosis of primitive cardiomyopathies, identification of cardiac involvement in systemic diseases, as well as risk stratification and monitoring of treatment effects in patients with heart failure of various etiologies [41]. In the adult CHD population, many of whom have had multiple previous surgeries and scars, the potential advantage of FT-CMR is that it can deliver quantification of myocardial deformation of the right ventricle, overcoming the limitation of the acoustic window [24].

The longitudinal systolic strain of RV and LV was assessed using CMR FT. RV strain for our cohort was impaired when compared with published CMR strain values from healthy volunteers (age 48 ± 13 years) [13], with significant correlation with RVEF, but no correlation with the extent of RV fibrosis (*p* = 0.844). RV EF is impaired decades after repair surgeries and RV FT-CMR analysis seems to mirror the effect of chronic pressure and/or volume load to RV, although without the ability to guide us regarding the RV performance and the adverse outcomes in this population.

A significant impairment of all global strain parameters with echocardiography-based 2D strain analyses has been described in rToF patients who experienced death or sustained ventricular tachycardia [25]. Numerous studies have attempted to determine the best values of echo-based RV GLS with reduced CMR RV EF in children and young adults with rToF in order to predict major cardiovascular events [26,42,43,44,45]. A recent study in adults with rToF and 2D STE study found that reduced RV myocardial strain is associated with worse outcomes [46]. On the contrary, Jing et al. state that FT-CMR parameters were not predictive indicators of the progressive dilation of the RV and therefore of any complications [47]. In our small study population, no significant correlation was revealed between the extent of RV fibrosis and RV GLS values. However, larger studies are needed to reach more robust conclusions.

LV longitudinal systolic analysis showed that LV systolic dysfunction coexists with relatively preserved LV EF in adults with rToF and impaired RV EF. Although focal fibrosis was not detected in the LV in the present study, LV performance was affected, with a statistically significant difference between groups of patients with maximum and lower LGE RV scoring, indicative of adverse progressive ventricular–ventricular interaction [25,26]. Considering that the interventricular septum is mainly a constituent part of the LV and only contributes 20% to RV systolic performance [48], the grade of dilatation and dysfunction of RV in rToF can affect LV GLS values.

Adverse clinical outcomes in adult patients with rToF have been associated with LV dysfunction, with the reasons for left ventricular dysfunction poorly understood. LV displacement by 2D STE study, in a study from Diller et al. [49] with 413 adult patients with rToF, showed that LV longitudinal dysfunction was associated with a higher risk of sudden cardiac death/life-threatening ventricular arrhythmias.

The novelty of our study is the correlation of the estimated focal RV fibrosis with the LV strain values and RV deterioration. A previous study of Hagdorn et al. in younger adults with rToF (median age 24.3 years, 54 patients <18 years) reported that LV GLS values predict ventricular tachyarrhythmia, but not deterioration of ventricular function [50]. A recently published study of de Alba et al. in 48 rTOF subjects and 20 healthy controls reported no association between LV global systolic strain and LV diffuse interstitial myocardial fibrosis (LV-ECV) evaluated by T1 mapping [51]. What seems certain is that changes in RV size and function lead to the asynchronization of the LV and in adverse cases inter-ventricular dysfunction [39,40]. Studies have shown that the amount of myocardial fibrosis affects the function of the LV and patients who underwent corrective surgery at an older age had a higher risk of developing LV dysfunction [52,53]. Our study confirms that CMR-FT reveals LV dysfunction in adults with rToF despite normal LVEF, due to decreased fibrotic RV performance.

Larger prospective studies are needed with techniques such as CMR-FT, which are reproducible, quick, and easy to apply, in order to establish the prognostic role of biventricular GLS in risk stratification of the rToF aging population.

### 4.3. CMR-FT Analysis and Biomarkers Levels in Adults with rToF

Despite the perception of many that TOF is a mainly right-sided heart disease, various LV parameters have been associated with this outcome [40,41]. Therefore, the NTproBNP levels and LV GLS values could be useful and sensitive indicators of early recognition of fibrotic RV performance disorders in this special population.

**Figure 3:**
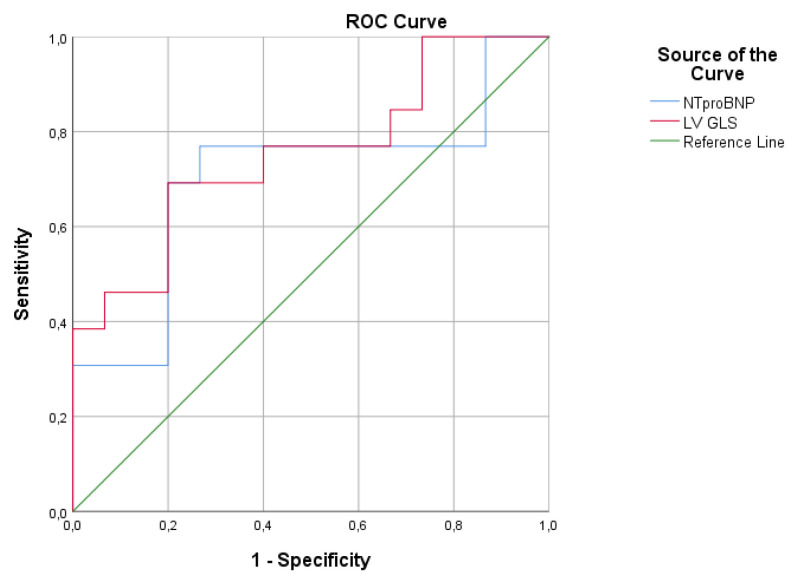
In ROC curve analysis, a cut-off value for NTproBNP of 145.25 pg/mL predicted LGE RV score > 8 points with specificity 80% and sensitivity 73.3% [AUC 0.729 (*p* = 0.03)], and a cut-off value for LV GLS of −15.9% predicted LGE RV score > 8 points with specificity 80% and sensitivity 69.2% [AUC 0.754 (*p* = 0.02)].

We found that longitudinal strain analysis by CMR-FT of the LV was impaired in adults with rToF, despite the preserved LV EF and correlation with high RV fibrosis score. According to the normal values for ages <75 years old of the non-HF population (<125 pg/mL), mean values of NTproBNP levels in our study were increased (181.2 ± 178.4 pg/mL), with preserved LV EF. NTproBNP levels correlated significantly with high RV fibrosis score. In ROC curve analysis, a cut-off value of NTproBNP > 145pg/mL and a cut-off value of GLS LV −15.9% predicted LGE RV >8 points, reflecting the coexistence of the LV longitudinal systolic dysfunction due to ventricular–ventricular interaction and neurohormonal activation, as result of high RV fibrosis (Figure 3).

We assume that further studies of LV GLS analysis and NTproBNP levels could contribute to the identification of early intervention time and risk stratification of adults with rToF.

### 4.4. Limitations

Our study has some limitations. The cohort of patients analyzed in this study does not reflect the entire spectrum of adult patients with rToF, as patients unsuitable for CMR assessment with gadolinium, including the higher risk patients with implantable cardioverter defibrillators, were excluded.

Finally, we used the late gadolinium technique to assess the extent of regional myocardial fibrosis on CMR. T1 mapping sequences were not available in our site and therefore the assessment of diffuse myocardial fibrosis was not possible.

A larger, prospectively followed-up cohort may provide further information on additional causes of fibrosis, its correlation with novel methods of RV and LV performance, as well as its prognostic power.

## 5. Conclusions

A significant extent of RV fibrosis was mainly detected at surgical sites of RV. Gal-3, could serve as a blood collagen biomarker for the noninvasive assessment of volume overload due to significant PR in adults with rToF. RV fibrosis leads to impaired LV performance and possible neurohormonal activation. Non-invasive imaging with CMR, with the detection of myocardial fibrosis and strain analysis, could allow for a more refined timing of interventions such as PVR. A larger, prospectively followed-up cohort may provide further information concerning additional causes of RV fibrosis, the correlation of biomarker levels and strain analysis with CMR-FT, and their precise prognostic value.

## Figures and Tables

**Table 1:** Demographics, clinical characteristics, CMR data and biomarker levels of 35 adult patients with rToF *.

	Median (SD) or N (%)
Demographics	
Age (years)	31.48 (10.8)
Age at ToF Repair (years)	1.8 (3.04)
Gender (male)	14 (40%)
Body surface area, (m^2^)	1.80 (0.20)
Height (cm)	168.6 (9.5)
Weight (kg)	71.34 (16.2)
Surgical History	
ToF repair	15 (57.7%)
ToF repair + PVR	11 (42.3%)
B-Tshunt + ToF repair	3 (33.3%)
B-T shunt + ToF repair + PVR	6 (66.7%)
Maximum number of surgeries (Nr ≥ 3)	6 (17.1%)
Clinical Data	
New York Heart Association class > II	25 (71.5%)
Rest Oxygen saturation > 96%	31 (88.5%)
QRS duration, (ms)	130.8 (18.8)
CMR	
RV EDVi, mL/m^2^	115.4 (35.5)
RV ESVi, mL/m^2^	55.6 (26.4)
RV EF, (%)	49.4 (8.2)
RV GLS, (%)	−20.8 (2.4)
LV EF, (%)	58.7 (6.0)
LV GLS, (%)	−17.04 (2.6)
Pulmonary regurgitation fraction, (%)	21.1 (17.1)
PR Fraction > 20%	16 (45.7%)
Biomarkers	
Galectin 3, (ng/mL)	6.4 (1.57)
Procollagen III, (ng/mL)	43.8 (11.1)
NTproBNP, (pg/mL)	181.28 (178.4)

* Continuous variables are expressed as mean (SD). Categorical variables are expressed as N (%). TOF, tetralogy of Fallot; B-T shunt, Blalock–Taussig shunt; PVR, pulmonary valve replacement.

**Table 2:** Association of RV LGE to clinical markers, ventricular volumes, biventricular performance by CMR and blood biomarkers *.

	Low RV Score LGE RV < 8 Points (*n* = 15)	High RV Score LGE RV ≥ 8 Points (*n* = 20)	*p* Value	Total RV Score Spearman Correlation Coefficient, (*p*)
Age, (years)	30.4 (11.6)	33.8 (10.7)	0.40	0.24 (0.170)
Age at repair, (years)	0.43 (0.37)	0.35 (0.30)	0.54	−0.02 (0.916)
Shunt to repair time (years)	1.9 (3.9)	1.3 (2.6)	0.67	0.15 (0.416)
Follow-up since repair, (years)	25.1 (10.9)	27.5(8.4)	0.52	0.27 (0.152)
Clinical				
NYHA Class ≥ II, [N, (%)]	9 (60%)	12 (60%)	0.21	-----
Rest SatO2, (%)	97.6 (1.1)	96.5 (1.8)	0.05	−0.46 (0.012)
QRS duration, (ms)	134.1 (17.6)	130.0 (20.3)	0.56	−0.18 (0.328)
Cardiac Magnetic Resonance				
RV EDVi, mL/m^2^	101.39 (19.8)	134.61 (43.1)	0.017	0.44 (0.015)
RV ESVi, mL/m^2^	46.0 (12.4)	73.4 (27.0)	0.003	0.66 (<0.001)
RV EF, (%)	53.4 (4.4)	43.3 (8.1)	0.001	−0.69 (<0.001)
RV GLS, (%)	−20.75 (2.3)	−20.75 (2.7)	0.99	−0.03 (0.844)
LV EF, (%)	61.1 (5.3)	56.5 (6.3)	0.04	−0.46 (0.011)
LV GLS, (%)	−18.0 (2.6)	−15.9 (2.1)	0.03	0.49 (0.007)
PR Fraction, (%)	16.2 (16.0)	24.6 (18.0)	0.20	0.44 (0.017)
Biomarker Levels				
Galectin 3, (ng/mL)	5.9 (1.33)	6.8 (1.64)	0.10	0.23 (0.211)
Procollagen III, (ng/mL)	42.5 (8.06)	43.5 (13.0)	0.78	−0.28 (0.123)
NTproBNP, (pg/mL)	121.6 (70.3)	196.4 (99.9)	0.02	0.29 (0.110)

* Continuous variables are expressed as mean (SD). Categorical variables are expressed as N (%). Statistical significance was defined as *p* < 0.05. CMR, cardiac magnetic resonance; RV, right ventricular; EDV, end-diastolic volume; ESV, end-systolic volume; EF, ejection fraction, GLS, Global Longitudinal Strain.

## Data Availability

The data presented in this study are available on request from the corresponding author. All the data are stored at the local research server and are not publicly available.

## Supplementary Materials

The following are available online at https://www.mdpi.com/article/10.3390/diagnostics11112101/s1, Table S1: CMR LGE RV and LV scoring, Figure S1: Distribution of LGE RV scores, Figure S2: Frequency of LGE RV detection according to the segmentation system of RV (%), Figure S3: Effect of LGE RV extent and severity of PR in RVEF% in adults with rToF, Table S2: Relations of Biomarkers levels with the severity of residual PR.
