# Supplementary material for: The Interplay between Myocardial Fibrosis, Strain Imaging and Collagen Biomarkers in Adults with Repaired Tetralogy of Fallot
Source: Diagnostics (Basel). 2021 Nov 13;11(11):2101. doi: 10.3390/diagnostics11112101 (PMC8621125; doi:10.3390/diagnostics11112101)
Supplement: Supplementary file 1 [file diagnostics-11-02101-s001.zip › diagnostics-1416410-supplementary/Diagnostics Supplementary Materials_Karali.pdf]

Supplementary Materials

Supplementary Table S1: CMR LGE RV and LV scoring.

**LGE score (points)**		**Mean (SD)**	**Median (IQR)**
LGE RV			
	RVOT scar area	2.5 (0.8)	3 (2, 3)
	Anterior wall of RV	1.09 (0.8)	1 (0, 2)
	Inferior wall of RV	0.2 (0.4)	0 (0, 0)
	RV surface of septum	0.7 (0.6)	1 (0, 1)
	VSD patch region	1.16 (1.2)	1 (0, 2)
	Trabecular bands	0.3 (0.4)	0 (0, 1)
	RV insertion points	1.3 (0.4)	1 (1, 2)
	Total LGE RV score	7.4 (2.8)	8 (6, 10)
LGE LV	LV surface of septum	0.0 (0)	0 (0, 0)

** Continuous variables are expressed as mean (SD) and median (IQR). RV, right ventricular; LGE, Late gadolinium enhancement; LV, left ventricular; RVOT, right ventricular outflow tract; VSD, ventricular septal defect.*

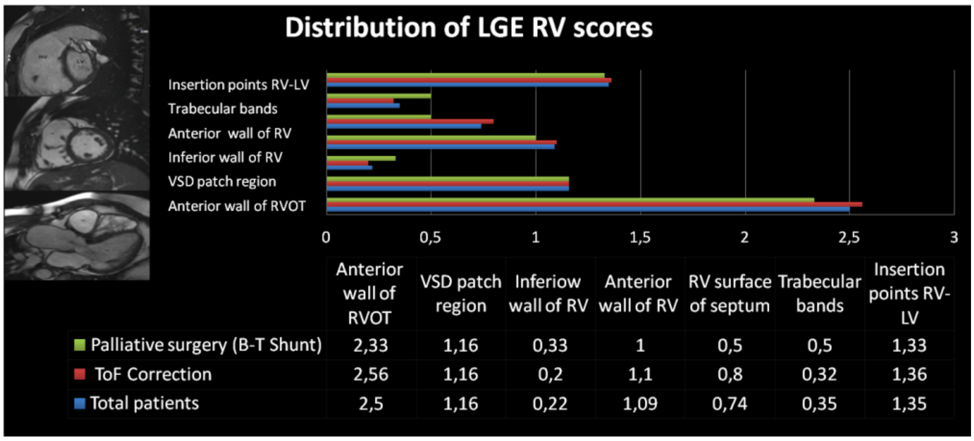

Supplementary Figure S1: Distribution of LGE RV scores. (Mean values of LGE RV scores).

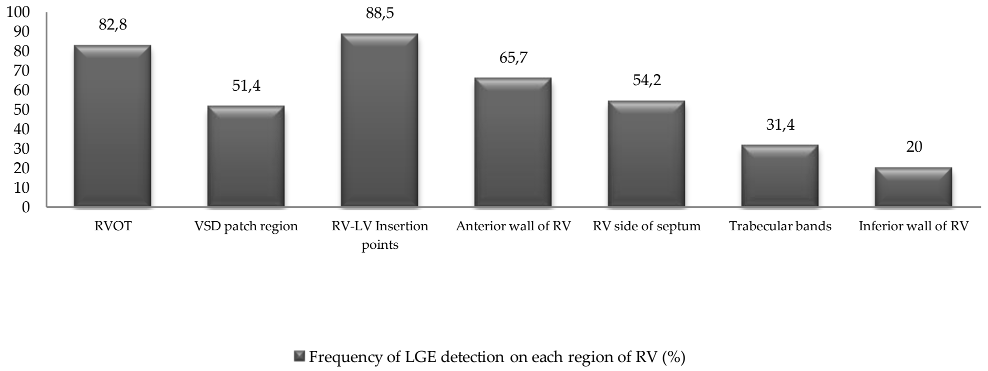

Supplementary Figure S2: Frequency of LGE RV detection according the segmentation system of RV (%).

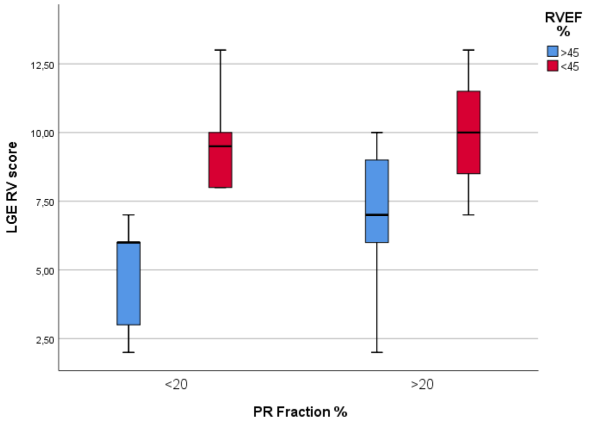

Supplementary Figure S3: Effect of LGE RV extent and severity of PR in RVEF% in adults with rToF.

**LGE, Late gadolinium enhancement; RV, right ventricular; EF, ejection fraction; GLS, Global Longitudinal Strain; PR, pulmonary regurgitation; LV, left ventricular.*

Supplementary Table S2: Relations of Biomarkers levels with the severity of residual PR.

	PR Fraction < 20% Mean (SD)	PR Fraction > 20% Mean (SD)	p value
Gal-3, (ng/ml)	5.9 (1.13)	7.1 (1.84)	**0.044**
PCIII, (ng/ml)	43.4 (6.97)	43.6 (13.6)	0.961
NTproBNP, (pg/ml)	136.5 (85.8)	223.3 (245.07)	0.206

** Continuous variables are expressed as mean (SD). Statistical significance was defined as $p < 0.05$. Significant results are highlighted in bold. PR, pulmonary regurgitation.*

Supplementary Table S3: RV LGE groups according to the surgical history of study population.

	**Low RV score**<br><br>**LGE RV < 8 points (n=15)**	**High RV score**<br><br>**LGE RV ≥ 8 points (n=20)**	***p* value**
First palliation: B-T shunt	4 (26.7)	4 (20)	0.394
First palliation: Total ToF repair	11 (73.3)	16 (80)	
Max number of surgeries, (Nr≥3)	2 (13.3)	2 (10)	1.0
PVR after repair	4 (26.7)	6 (30)	0.704

** Categorical variables are expressed as N (%). TOF, tetralogy of Fallot; B-T shunt, Blalock–Taussig shunt; PVR, pulmonary valve replacement.*
