# Supplementary figures and images for: The Interplay between Myocardial Fibrosis, Strain Imaging and Collagen Biomarkers in Adults with Repaired Tetralogy of Fallot
Source: Diagnostics (Basel). 2021 Nov 13;11(11):2101. doi: 10.3390/diagnostics11112101 (PMC8621125; doi:10.3390/diagnostics11112101)

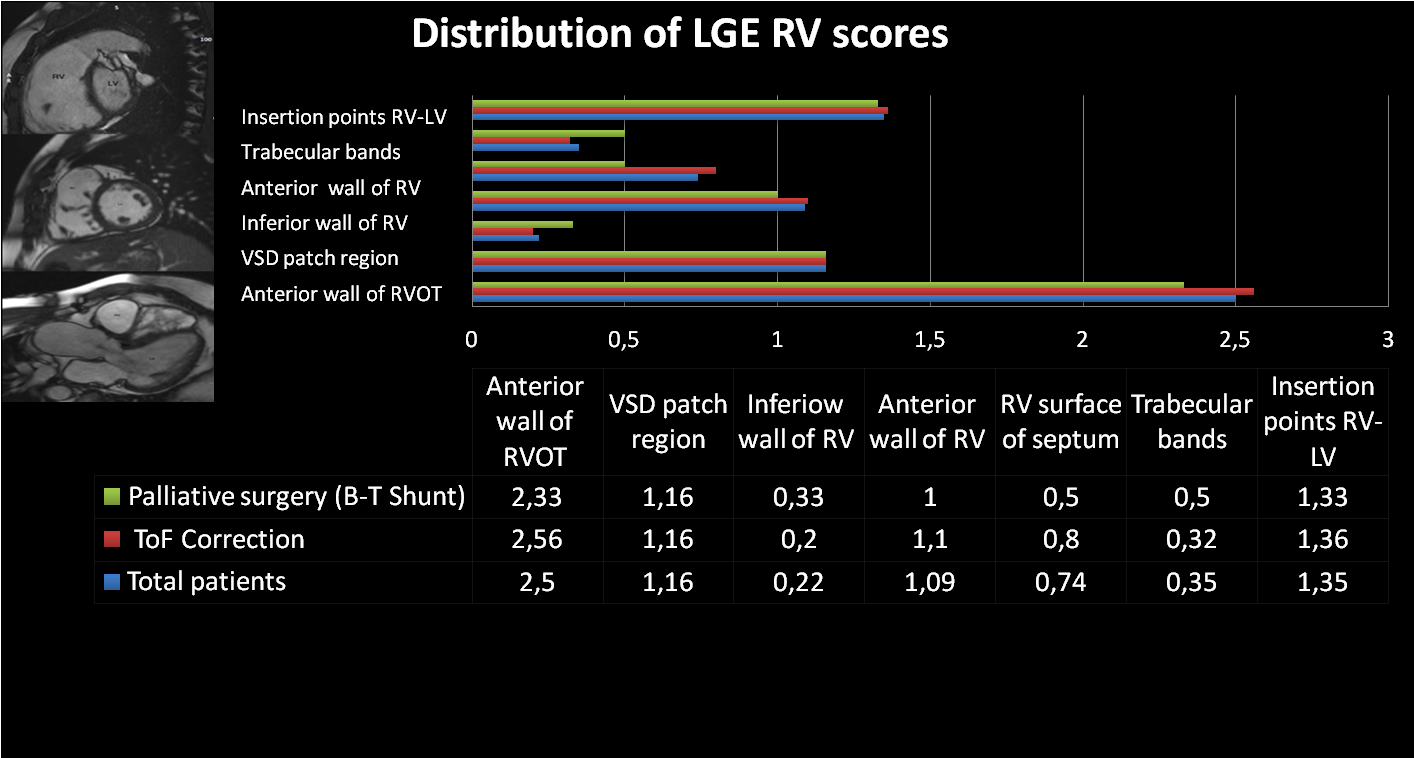

Supplement: Supplementary file 1 [file diagnostics-11-02101-s001.zip › diagnostics-1416410-supplementary/Supplementary Figure S1.tif]

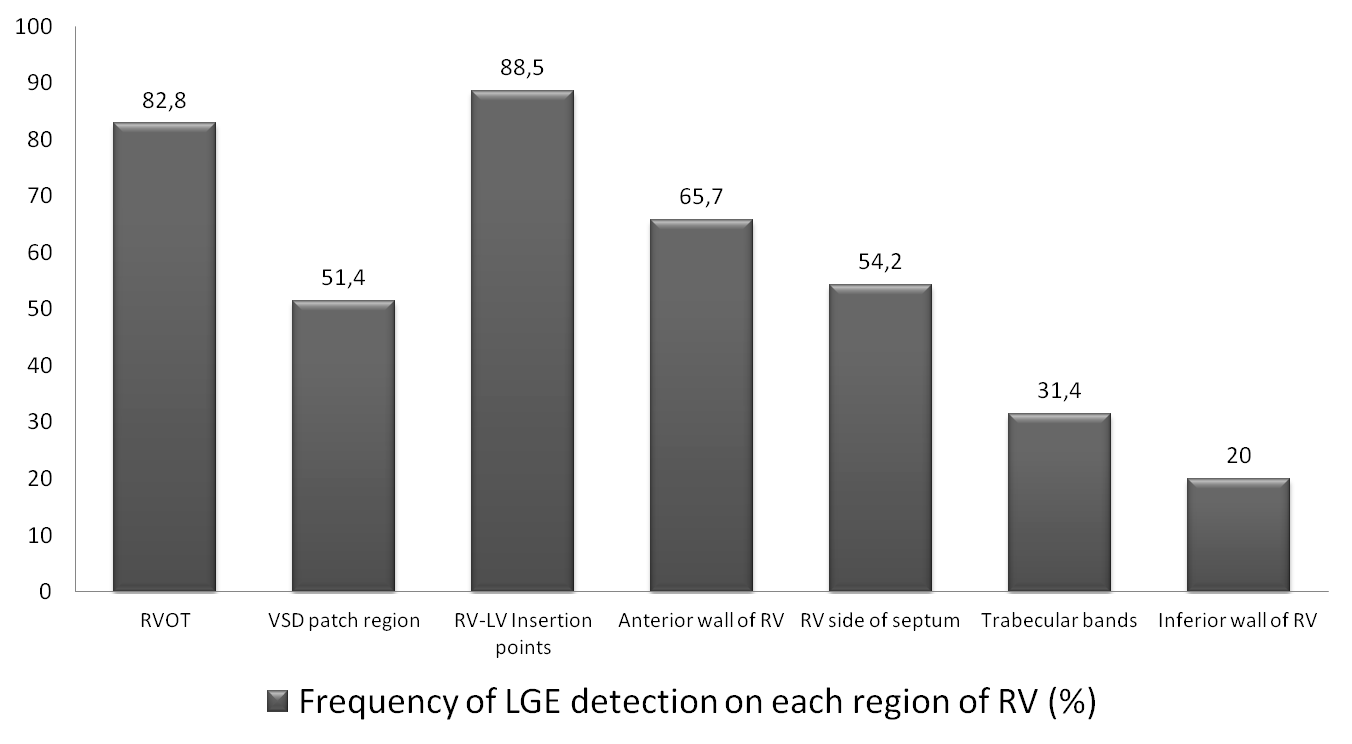

Supplement: Supplementary file 1 [file diagnostics-11-02101-s001.zip › diagnostics-1416410-supplementary/Supplementary Figure S2.tif]

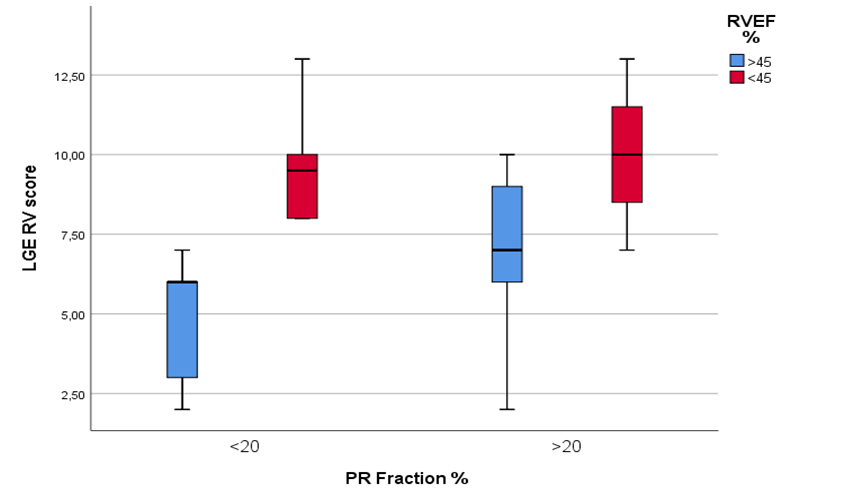

Supplement: Supplementary file 1 [file diagnostics-11-02101-s001.zip › diagnostics-1416410-supplementary/Supplementary Figure S3.tif]
